# Cross-cultural adaptation and validation of Sarcopenia and Quality of Life (SarQoL) in Brazil

**DOI:** 10.1590/1516-3180.2021.0968.R1.07042022

**Published:** 2022-08-29

**Authors:** Fabiana de Souza Orlandi, Juliana Duarte Nunes, Diana Gabriela Mendes dos Santos, Aline Cristina Martins Gratão, Marisa Silvana Zazzetta

**Affiliations:** I PhD. Associate Professor, Department of Gerontology, Universidade Federal de São Carlos (UFSCar), São Carlos (SP), Brazil.; II MD, MSc. Gerontologist, Department of Gerontology, Universidade Federal de São Carlos (UFSCar), São Carlos (SP), Brazil.; III MD, MSc. Gerontologist, Department of Gerontology, Universidade Federal de São Carlos (UFSCar), São Carlos (SP), Brazil.; IV PhD, Adjunct Professor, Department of Gerontology, Universidade Federal de São Carlos (UFSCar), São Carlos (SP), Brazil.; V PhD, Associate Professor, Department of Gerontology, Universidade Federal de São Carlos (UFSCar), São Carlos (SP), Brazil.

**Keywords:** Sarcopenia, Aged, Validation study, Quality of life, Translations, Translation and adaptation, Older adults, Life quality

## Abstract

**BACKGROUND:**

Sarcopenia is defined as a slow, progressive, and apparently inevitable process of involuntary loss of muscle mass, strength, and quality, which occurs with advancing age. It is widely accepted that sarcopenia can directly affect quality of life.

**OBJECTIVE:**

Translate, adapt and validate the “Sarcopenia and Quality of Life” instrument (SarQoL) to the Brazilian context.

**DESIGN AND SETTINGS:**

Translation, cross-cultural adaptation, and validation study carried out at the Federal University of São Carlos, São Carlos, São Paulo, Brazil.

**METHODS:**

The population consisted of 221 older adult participants. The steps recommended by the guidelines from the authors of the original instrument were followed sequentially: initial translation, synthesis of translations, backward translation, evaluation by a panel of judges, pre-test, and analysis of psychometric properties. The translation and adaptation process was conducted as recommended.

**RESULTS:**

Two hundred and twenty-one participants took part in the step analysis of the psychometric properties of SarQoL, in which 55 presented sarcopenia. Cronbach's alpha coefficient of the total SarQoL questionnaire was 0.976, indicating excellent internal consistency. Excellent agreements between the test and retest with an Interclass Correlation Coefficient (ICC) of 0.983 (95% confidence interval: 0.901–0.996) were observed in the SarQoL domains. The domains of Short-Form 36 and EuroQoL 5-dimension showed significant correlation, from moderate to strong magnitude, with SarQoL total score, indicating convergent validity.

**CONCLUSION:**

The Brazilian version of SarQoL presented evidence of reliability and validity.

## INTRODUCTION

The elderly population in Brazil is rapidly increasing and is currently ranked sixth largest in the world, with more than 23 million citizens over 60 years of age, accounting for 12.6% of the Brazilian population.^1^ Due to this accelerated growth, there is a considerable increase in chronic conditions related to age, such as sarcopenia, attracting the attention of many researchers in this field.^2^

Sarcopenia is defined as a slow, progressive, and seemingly inevitable process of involuntary loss of muscle mass, strength, and quality, which occurs with advanced age.^3,4^ To diagnose sarcopenia, according to the 2^nd^ European Working Group on Sarcopenia in Older People (EWGSOP2), if low muscle strength is detected, confirm sarcopenia through the amount of muscle mass and to classify it as severe sarcopenia, check physical performance.^5^

Skeletal muscle dysfunction is a debilitating condition that leads to daily limitations.^6^ Muscle mass, strength, and physical performance are particularly determinant of independent function in human life.^6–8^

In a meta-analysis, sarcopenia was found to be an independent risk factor for several adverse outcomes, including dependence for instrumental and basic activities of daily living, osteoporosis, hospitalization, and death.^8^ It is therefore widely accepted that sarcopenia can directly impair quality of life.^6–9^

To evaluate the quality of life, specifically of people with sarcopenia, Sarcopenia and Quality of Life (SarQoL) was developed and validated in Belgium.^9,10^ It consists of seven domains: physical and mental health, locomotion, body composition, functionality, activities of daily living, leisure activities, and fears.^9^ Currently, SarQoL has been translated into 26 other languages including Dutch, German, Spanish, Italian, Greek, Hungarian, Romanian, Ukrainian, Polish, Persian, and Czech. It should be mentioned that the French, English, Polish, Romanian, Dutch, Greek, and Lithuanian versions have already been validated.^11^

A systematic review and meta-analysis on the prevalence of sarcopenia in Brazilian older adults returned 31 completed studies with more than nine thousand older adult patients. Results showed that 17% of older adult patients present sarcopenia,^2^ meaning that instruments for assessing the specific quality of life of this group are necessary.

## OBJECTIVE

To translate, culturally adapt and validate the “SarQoL” instrument in the Brazilian context.

## METHODS

### Ethical considerations

This study was approved by the Research Ethics Committee of the Universidade Federal de São Carlos (UFSCar), under opinion number 637.779 / 2016 on July 14, 2016.

### Procedures

#### Translation, adaptation, and validity

SarQoL has 55 items in 22 questions and includes seven domains. Its score ranges from 0 to 100, and the higher the score, the better the quality of life.^9^ SarQoL is a simple, easy-to-use instrument and takes an average time of 10 minutes to complete. It can also be self-applied.^9–11^

It should be noted that prior to initiating the SarQoL translation, adaptation, and validation process, the authors′ permission was obtained by e-mail (Charlotte Beaudart and Olivier Bruyère- www.sarqol.org).

The following recommended steps were followed according to the protocol provided by the authors who designed the original instrument:

- Initial translation: This was conducted by two qualified and independent translators. One had a medical background and the other did not have any specialized knowledge on the subject of the questionnaire. The translation by two qualified and independent translators allows for the detection of errors resulting from divergent interpretations of ambiguous terms in the original language. Both translators provided a written report with commentary in order to highlight phrases or uncertainties and the reasons for the specific language choices made.
- Synthesis of the translations: Translators and researchers met to conduct a synthesis of the results of the translations and to establish a consensus in the face of the divergences found or ambiguous interpretations of the SarQoL.
- Back-translation: The translation into the original language was conducted by two other translators fluent in both languages and whose mother tongue is English. The translators were unaware of the study objectives. In addition, each translation was independently reverse engineered, allowing for error detection.
- Review Committee: A committee of judges reviewed and compared all translations conducted with the aim of producing a final version, modified and adapted to guarantee a replica of the instrument for use in Brazil. The committee comprised two methodologists, two health professionals, a Portuguese-speaking professional, and the four translators involved in the process. After the evaluation of this committee, the pre-final version of the SarQoL was obtained.
- Pre-final version test: The pre-final version in Portuguese of the SarQoL was performed with 20 older adults of different educational and socioeconomic levels, diagnosed with sarcopenia. Participants were asked about difficulties in completing the questionnaire or understanding the purpose or meaning of the questions. Following the interview process, the expert committee discussed the results and proposed the final version.
- Validation of psychometric properties: At this stage, 221 older adults from the community who accessed primary health care in the city of São Carlos, São Paulo, Brazil, were evaluated.

This was a cross-sectional study. The older adults were individually invited, through telephone contact, in which the objective and stages of the study were explained. After acceptance, a visit was scheduled at the older adult participant's residence, where the researchers collected data. Post signing the consent form, they answered the participant characterization questionnaire, SarQoL, EuroQoL-5D and Short Form Health Survey (SF-36), Geriatric Depression Scale, and mini nutritional assessment (MAN). After answering the aforementioned instruments, it was necessary to verify who among the older adult respondents were and were not sarcopenic.

To diagnose sarcopenia, the criteria recommended by the EWGSOP24 were adopted, in which muscle strength is assessed, sarcopenia is confirmed by muscle mass and severity determined by physical performance.^4^

As evidence of sarcopenia, the measurement of handgrip strength was used, in which the criterion established by the Health, Well-being and Aging (SABE) study was adopted, which uses the cut-off score of < 30 kg for men and < 20 kg for women.^13,14^ If participants had scores lower than those mentioned above, they qualified for the test of the first criterion (low strength).

To confirm sarcopenia by detecting low muscle quantity and quality, dual-energy x-ray absorptiometry (DXA) was used. After answering the aforementioned instruments, a day and time were scheduled for the participant to perform the DXA at the Physiotherapy Department of the UFSCar, where they were picked up at their own residence and taken back after taking the test. For the cut-off values, those recommended by the SABE study were used, that is, 6.37 m^2^/kg for women and 8.90 m^2^/kg for men.^15,13^

To determine the severity of sarcopenia, a gait speed test with a cut-off score of less than 0.8 m/s was used for both sexes.^3,5^ The speed test was conducted at the Physiotherapy Department of the UFSCar, in which the participant did the DXA and then performed the walk test in a prepared and controlled environment.

### Statistical analysis

The Kolmogorov-Smirnov test was first performed, to verify the absence of data normality. From this result, non-parametric statistical tests were adopted. In the descriptive analysis, the median values of the sociodemographic and health variables, as well as the frequency of the qualitative variables were determined. To analyze the reliability of SarQoL, the Cronbach's alpha coefficients, both for the total and the individual domains, were verified. Satisfactory internal consistency was considered for values equal to or greater than 0.7. To verify the test-retest reliability, the interclass correlation coefficient (ICC) was calculated for the total and individual SarQoL domains, with values equal to or greater than 0.7 indicating a satisfactory stability of the instrument.

For the SarQoL discriminant validity analysis between the group of the older adults with and without sarcopenia, the Mann-Whitney test was performed. To verify the discriminative power of SarQoL, a logistic regression analysis was conducted. The model was adjusted according to age and body mass index (BMI), which were the variables that presented a statistically significant difference between the groups (older adults with and without sarcopenia).

## RESULTS

This study satisfactorily implemented all the steps recommended by the original authors for the SarQol translation and adaptation process. A general agreement average of 95.5% was obtained in the analysis conducted by the expert committee and there was no semantic change in the pre-test phase.

Out of the 221 older adults evaluated in the study validation process, 55 (24.8%) participants had sarcopenia and 166 (75.1%) did not fulfill the criteria to diagnose sarcopenia. Female participants predominated the sample (n = 151, 68.3%), which had an average of four years of schooling, two daily medications, and four associated diseases.

In addition, only 31 older adults consumed alcoholic beverages (14.0%) and 19 were smokers (8.6%). Respondents with sarcopenia were older, average age of 73.2 years, compared to those without, who were on average 68.0 years old (P ≤ 0.001). Regarding the BMI, the older adults with sarcopenia had lower mean values than those without, with a BMI of 25.3 kg/m^2^ and 29.6 kg/m^2^, respectively (P ≤ 0.001). There was no difference in gender, schooling, number of associated diseases, number of medications in use, or consumption of alcoholic beverages and cigarettes between sarcopenic and non-sarcopenic participants.

### Reliability

Cronbach's alpha coefficient of the total SarQoL questionnaire was 0.976, indicating excellent internal consistency. Regarding the homogeneity of the domains of the SarQoL questionnaire, it can be observed that the values ranged from 0.622 to 0.976, also showing satisfactory internal consistency (Table 1).

**Table 1:** Results of the correlation between each domain and the total score of the SarQoL and of the test-retest reliability of the SarQoL total score and individual domain scores. n = 221

SarQoL	Correlation		Cronbach's Alpha	Test-Retest	
	r	P value		ICC	CI 95%
Total score			0.976	0.983	0.901–0.996
Physical and mental health	0.795	< 0.001	0.813	0.963	0.883–0.989
Locomotion	0.907	< 0.001	0.958	0.982	0.938–0.995
Body composition	0.647	< 0.001	0.845	0.990	0.966–0.997
Functionality	0.919	< 0.001	0.914	0.974	0.914–0.993
Activities of daily living	0.918	< 0.001	0.823	0.978	0.664–0.995
Leisure activities	0.540	< 0.001	0.615	0.930	0.774–0.979
Fears	0.599	< 0.001	0.735	1.000	1.000–1.000

SarQoL = sarcopenia and quality of life; ICC = interclass correlation coefficient; CI = confidence interval.

Table 1 shows that all domains correlated positively and significantly with the total SarQoL score. Excellent stability of the SarQoL verified through the ICC was also observed, using the test and retest of the questionnaire, in which the ICC of 0.983 (95% confidence interval, CI: 0.901–0.996) was observed in the SarQoL total and in all its domains as well.

### Discriminant validity

Table 2 shows that SarQoL was able to discriminate between the older adults with and without sarcopenia, in all domains of QoL, as well as the total SarQoL.

**Table 2:** Discriminative power of the SarQoL. n = 221

	Sarcopenia (n = 55)	Without Sarcopenia (n = 166)	P value*
Physical and mental health	62.85	75.44	**< 0.001**
Locomotion	50.94	70.82	**< 0.001**
Body composition	61.64	74.70	**< 0.001**
Functionality	61.81	76.52	**< 0.001**
Activities of daily living	46.05	73.71	**< 0.001**
Leisure activities	37.64	47.47	**0.001**
Fears	84.83	91.72	**0.001**
Total score	55.57	73.94	**< 0.001**

* Mann-Whitney Test.

SarQoL = sarcopenia and quality of life.

The older adults with sarcopenia had an average total score of 55.5 (± 18.67), compared to a score of 74.4 (± 18.06) in the older adults without sarcopenia. In the logistic regression model for the total SarQoL score between the groups, adjusted for age and BMI, the odds ratio (OR) of 0.963 (95% CI: 0.945–0.982) was obtained, indicating a low total score in participants with sarcopenia compared to those without. In addition, in the SarQoL domain regression analyses, participants with sarcopenia also had lower scores compared to the older adults without sarcopenia (Table 3). The discriminant power of the SarQoL questionnaire was confirmed.

**Table 3:** Regression analysis between participants with and without sarcopenia

	OR	CI 95%
Physical and mental health	0.964	0.948–0.981
Locomotion	0.966	0.953–0.979
Body composition	0.960	0.943–0.977
Functionality	0.949	0.931–0.968
Activities of daily living	0.951	0.935–0.967
Leisure activities	0.968	0.947–0.988
Fears	0.959	0.940–0.979
Total score	0.942	0.922–0.961

OR = odds ratio; CI = confidence interval.

### Convergent construct validity

Considering the validity of the convergent, it can be observed in Table 4 that all domains of the SF-36 and the visual analog scale present in the EQ-5D correlated positively and significantly with the total SarQoL score. Given these results, the convergent construct validity of the SarQoL is confirmed.

**Table 4:** Correlations of the total score of the SarQoL questionnaire with individual domains of the SarQoL, the SF-36 and the EQ-5D

		Spearman correlation coefficients	P value
Convergent validity			
	SF-36 Functional Capacity	0.852	< 0.001
	SF-36 Physical Aspects	0.558	< 0.001
	SF-36 Pain	0.607	< 0.001
	SF-36 General Health Condition	0.523	< 0.001
	SF-36 Vitality	0.572	< 0.001
	EQ-5D Mobility	−0.696	< 0.001
	EQ-5D Usual Activities	−0.719	< 0.001
	EQ-5D Utility Score	0.510	< 0.001

SarQoL = sarcopenia and quality of life; SF-36 = short form health survey.

## DISCUSSION

The present study satisfactorily implemented all the steps recommended by the original authors for the SarQoL translation, adaptation, and validation process in Brazil. SarQoL has been shown to be comprehensible, consistent, reliable, and valid, and therefore may be recommended for clinical and research purposes. The questionnaire is already available in 26 different languages, with more translations underway.^10,11^

Regarding internal consistency, it was observed that the SarQoL domains presented Cronbach's alpha coefficients between 0.62 and 0.97. In the SarQoL validation study in England, conducted with 235 older adults in the community-14 with sarcopenia and 221 without, Cronbach's alpha was found to be between 0.79 and 0.94,^12^ in line with the results of the present research. In the psychometric properties of the Spanish Version of SarQoL^16^ conducted with 252 older adults, 66 with sarcopenia and 186 without, Cronbach's alpha values were between 0.57 and 0.94, also compatible with the findings of the present investigation. Another study that corroborates these findings is the SarQoL validation study in Korean, carried out with 450 older adults in which 43.1% of the participants had sarcopenia, Cronbach's alpha was between 0.823 and 0.925.^17^

In the present study, excellent stability of SarQoL was observed, verified by the ICC, in which the ICC of 0.98 (95% CI: 0.90–0.99) was verified in the test-retest of the SarQoL total, as well as in all its domains. The data were corroborated with the validation study of the Dutch version of SarQoL, conducted with 92 older adults in the community, 30 of whom were sarcopenic, in which the ICC was 0.976 (95% CI: 0.947–0.989).^17^ Another study that corroborates this finding is the psychometric properties of the Spanish Version of SarQoL by Fábrega-Cuadros et al.,^16^ in which test-re-test data showed excellent reliability for the total Spanish SarQoL score ICC = 0.99 (95% CI: 0.98–0.99).

The Brazilian version of SarQoL was able to discriminate between the older adults with and without sarcopenia, in all domains, as well as in their total score. Specifically, in the total quality of life score of the Brazilian version of SarQoL, the older adults with sarcopenia and without sarcopenia had a total average of 55.5 and 73.9, respectively.

In the validation study of the Romanian version of SarQoL, conducted with 100 older adults in the community, 87 without sarcopenia and 13 with, the older adults with sarcopenia also had lower scores in all domains and in the total score, in which the sarcopenia respondents had a mean score of 57.3 (34.4–70.7) and the older adults without sarcopenia scored 68.4 (55.7–85.2).^18^ Data from the present study are also corroborated by results obtained from the validation of the SarQoL version in Dutch, in which the older adults with sarcopenia had a total average SarQoL score of 67.15 (54.75–81.52), lower than those without sarcopenia, who scored an average of 79.72 (70.10–86.88).^19^

Furthermore, in the SarQoL domain regression analysis, participants with sarcopenia displayed lower scores compared to the older adults without sarcopenia, corroborating the validation study in French: Physical and Mental Health (OR: 0.96; IC: 95%: 0.94–0.99), Locomotion (OR: 0.97, 95% CI: 0.95–0.98), Body Composition (OR: 0.95, 95% CI: 0.95–0, 99), Functionality (OR: 0.95, 95% CI: 0.93–0.98), Activities of daily living (OR: 0.93, 95% CI: 0.91–0.96), Leisure activities (OR: 0.97; 95% CI: 0.95–0.99) and Fears (OR: 0.95, 95% CI: 0.91–0.98).^11^

Regarding the convergent construct validity, it was observed in this study that the SF-36 and EQ-5D dimensions evaluated were significantly correlated with the total SarQoL score, corroborating the validation study of the English version of SarQoL, where they obtained the following results: Functional Capacity (r = 0.82, P < 0.001), Physical Aspects (r = 0.54, P < 0.001) (r = 0.55, P < 0.001), General Health Condition (r = 0.49, P < 0.001), Vitality (r = 0.74, P < 0.001), Mobility (r = −0.56, P < 0.001), Usual Activities (r = −0.55, P < 0.001) and Utility score: (r = 0.58, P < 0.001).^12^

In the validation study of the Turkish version of SarQoL, the authors found strong/good correlations between the total SarQoL-TR score and some SF-36 domains that have similar dimensions, such as physical functioning (r = 0.82, P < 0.001), vitality (r = 0.69, P < 0.001), function limitations due to physical problems (r = 0.69, P < 0.001) and general health (r = 0.60, P < 0.001).^20^ Strong/good correlations were also found between the total SarQoL-TR score and some domains of the EQ-5D that have similar dimensions, such as mobility (r = −0.59, P < 0.001), usual activities (r = −0.63, P < 0.001), self-care (r = −0.59, P < 0.001) and utility score (r = 0.77, P < 0.001).^20^ These findings are in line with the results of the present study in which all domains of the SF-36 and the visual analogue scale present in the EQ-5D correlated positively and significantly with the total score of the SarQoL.

The convergent validity of SarQoL Dutch version was also confirmed, obtaining the following results in the correlations with the SarQoL total score: Functional Capacity (r = 0.84, P < 0.001), General Health Condition (r = 0.62, P < 0.001), vitality (r = 0.65, P < 0.001), usual activities (r = −0.57, P < 0.001), Utility score (r = 0.47, P = 0.002), in addition to the Mobility-test questionnaire (r = 0.77, P < 0.001).^21^

In a study correlating SarQoL with 4937 older adults in Korea through DXA and EQ-5D, they found that 6.6% of the evaluated older adults presented with sarcopenia and showed greater losses in all the domains of the EQ-5D, demonstrating that the condition directly influences the quality of life.^17^

A limitation of the study is the sensitivity to change of SarQoL, which will need to be evaluated in future longitudinal and clinical intervention studies.

## CONCLUSION

Based on the proposed objective and results obtained, it can be concluded that the SarQoL shows evidence of reliability and validity. SarQoL is translated, adapted, and validated in the Brazilian context, and is available for use in Brazil (www.sarqol.org).
